# The role of socio-economic inequality in the prevalence of hypertension in adults

**DOI:** 10.15171/jcvtr.2019.20

**Published:** 2019-06-30

**Authors:** Yousef Veisani, Ensiyeh Jenabi, Shahrzad Nematollahi, Ali Delpisheh, Salman Khazaei

**Affiliations:** ^1^Psychosocial Injuries Research Center, Ilam University of Medical Sciences, Ilam, Iran; ^2^Autism Spectrum Disorders Research center, Hamadan University of Medical Sciences, Hamadan, Iran; ^3^Men’s Health and Reproductive Health Research Center, Shahid Beheshti University of Medical Sciences, Tehran,Iran; ^4^Department of Clinical Epidemiology, Ilam University of Medical Sciences, Ilam, Iran; ^5^Research Center for Health Sciences, Hamadan University of Medical Sciences, Hamadan, Iran

**Keywords:** Hypertension, Prevalence, Socioeconomic Factors, Inequality

## Abstract

***Introduction:*** The large portion of burden of diseases, especially in the developing countries is attributed to hypertension. Identification of the potential risk factors of hypertension is essential for disease management. In this study we investigated the role of socio-economic inequality in the prevalence of hypertension in Ilam Province.

***Methods:*** Totally, 690 individuals aged over 15 were enrolled in this cross-sectional study, through systematic random sampling from March 1 to October 30, 2017. Socio-economic status (SES) score was calculated by 7 variables including; age, sex, job, marital status, educational level, and economic status, residency, then, it was divided to five levels. Concentration index was used to estimate the inequality in hypertension. To estimate the percentage contribution in final step elasticity divided to concentration index for each contributor and contributions to inequality is estimated.

***Results:*** The concentration index for hypertension was -0.154 95% CI (-0.02, -0.23), therefore hypertension was more prevalent in lower socioeconomic groups. The important socioeconomic contributors in inequality were job (*P*=0.008), educational level (*P*=0.005), and SES (*P*=0.003). According to concentration index decomposition, the main sources of inequality in hypertension were job (15%), educational level (18%), and SES (21%), respectively.

***Conclusion:*** Hypertension is more prevalent in lower SES groups and the job, education, and SES are important contributory factors of inequality. One substantial key point to achieve an effectiveness approach to deal with chronic diseases might be building partnership with disadvantaged populations.

## Introduction

According to estimations, the prevalence of hypertension will be 29.2% in 2025 in the world and 60% in adults.^1^ Cardiovascular diseases and stroke are the consequences of hypertension^2^ and the lower and middle-income societies are the victims of more than 80% of deaths from cardiovascular diseases.^3^ Socio-economic status (SES) can be considered as a necessary factor for health based on lots of evidence; however, a few studies associated with SES have been conducted that were measured by the individual and country levels and cardiovascular risk factors among society.^4^

The relationship between high blood pressure and low-level socio-economic condition was shown in the study by Riva et al in 2016.^5^ According to a study conducted in Iran in 2014, there was more prevalence of hypertension in low SES individuals. Moreover, women and urban residents had a greater effect to the increase of the inequality.^6^ Education, income, employment and housing status are used to evaluate the SES in the individuals. In non-indigenous populations, there is an association between SES and better health outcomes.^4^ Leng et alin a meta-analysis showed that in the lowest SES in income, job and education, the prevalence of hypertension was higher than the highest SES. High-income countries showed a significant association and women showed high risk of hypertension for the lowest categories of all SES indicators, while men showed less consistent association.^7^

Based on some studies, reducing socio-economic inequality can be successful by identifying these causes and empowering them in material, psychosocial, and political dimensions. So, this study aims at investigating socio-economic inequality in the prevalence of hypertension in Ilam province.^8^

## Materials and Methods

Totally, 690 individuals aged over 15 were enrolled in this cross-sectional study, from March 1 to October 30, 2017. All participants were recruited by systematic random sampling in 7 comprehensive healthcare centers in Ilam city, the capital of Ilam province. The proportion of the subjects in each center was obtained by adjusting them to the population of the same area, following systematic recruitment in the study. The inclusion criteria in this study was (1) Age 15 or older, (2) having informed consent to participate in the study, and (3) lack of any physical and mental illnesses. The exclusion criteria was (1) non-readable response to the questionnaire, and (2) opt out of participation in the study.

In the current study, hypertension was the outcome variable and the other independent variables were age (15-25 years old; 25–44 years old; 45–64 years old; and >65 years old), sex (male/female), educational level (illiterate, primary school, guide/high school, diploma, and university), residence (urban/rural), job status (unemployment and employed), marital status (married and single), and economic status (low, middle, and high). Also, principal component analysis (PCA) was used to calculate the SES variable and subjects were classified to five levels (poorest, second, middle, fourth, and richest) to estimate the prevalence of hypertension in different levels. In PCA procedure, the included variables that had greater impact on the whole variance of SES were identified. A dummy variable was created for nominal variables such as residence (rural) and job status (unemployment) and a total of 7 variables enrolled in final model. In the final step, new variables of SES were identified in 5 groups.

### Statistical analysis

SES score was calculated by 7 variables including; age, sex, job, marital status, educational level, and economic status, residency, then it was divided to five levels. A logistic regression model was used for assessment of the variables on hypertension.

Concentration Index was used to estimate the inequality of hypertension. Concentration index equals to zero corresponds to no inequality, while positive and negative concentration index indicating that hypertension is more concentrated in high and low socio-economic groups.

Decomposing of concentration index revealed important contributors in inequality. In decomposing process, the variance of changes by each included variable calculated as elasticity by βkXk/µ formula, that there X=x_1_, x_2_… x_k_ and µ is the mean score of hypertension prevalence. Afterwards, absolute contribution to inequality by each contributor was estimated by (βkXk/μ) Ck Formula, Here, Ck refers to concentration index for contributors. The percentage contributions to inequality was obtained through elasticity divided to concentration index in each category (βkXk/µ)Ck/. In this study, the igini, clorenz and rbdineq commands were used by Stata software version 11.2 (StataCorp, College Station, TX, USA). Data was analyzed at 0.05% significance level.

## Results

**Figure 1:**
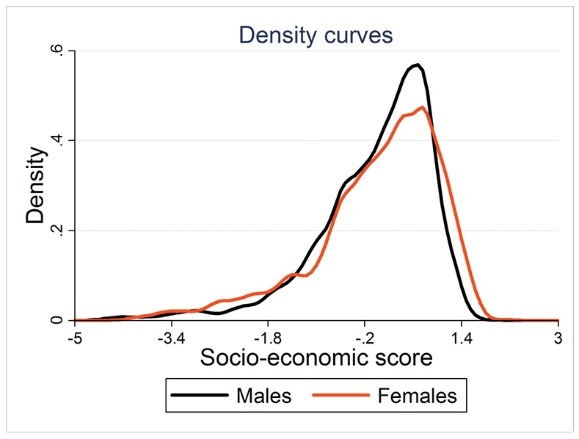
Distribution of socio-economic scores.

**Figure 2:**
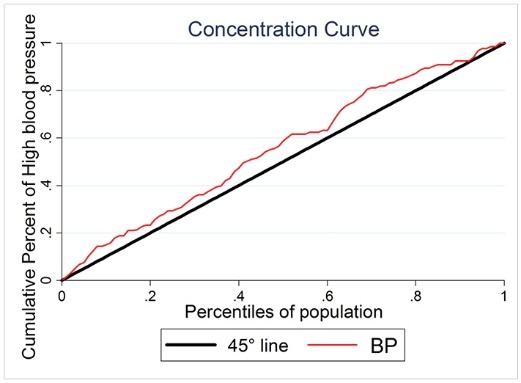
High blood pressure ranked by socio-economic status in all participants.

Socio-economic score was calculated for all the participants (aged from 15-90) (N= 690). Density curve by socio-economic scores for males and females was shown in Figure 1. The distribution of scores in males and females tends to skewed to the left, therefore, the proportion of the subjects belonged to high socio-economic groups including; 33.19% in the middle, 32.7% in the fourth, and 15.36% in the richest. Inequality line by concentration curve was drawn based on High blood pressure in the subjects. The concentration index for hypertension was -0.154 95% CI [-0.02, -0.23], therefore hypertension was more prevalent in lower socio- economic groups (Figure 2).

Logistic regression analysis was conducted for predicting related factors in hypertension. Accordingly, the odds of hypertension were increased with increasing age. The odds of hypertension in the age group of ≥66 was higher than the age group of 15-24 (odds ratio [OR]=1.89, 95% CI: 1.19, 2.87). The odds of hypertension were higher in males compared to females (OR=1.21, 95% CI: 1.01, 3.36). The OR for residency indicated that urban subjects were associated with 30% increase in the odds of developing hypertension compared to rural ones (OR=1.30, 95% CI: 1.05, 3.59). Other results of the employed subjects showed that the employed subjects had decreased odds of hypertension compared to the subjects who were unemployment (OR=0.66, 95% CI: 0.47, 0.87). Also, it was found that the subjects with academic degree had less risk of hypertension compared to the illiterate subjects (OR=0.72, 95% CI: 0.68, 0.97). It was observed that single individuals had a lower risk of hypertension compared to the married ones (OR=0.91, 95% CI: 0.63, 0.84) (Table 1).

**Table 1:** Logistic regression analyses of predicting factors in high blood pressure

**Independent variables**	**Adjusted OR**	**95% CI**	***P*** ** value**
Age group (y)			
15-24	Reference	1	
25-44	1.36	1.21-1.60	<0.001
45-64	1.59	1.11-2.16	<0.001
≥65	1.89	1.19-2.87	<0.001
Sex			
Female	Reference	1	
Male	1.21	1.01-3.36	0.012
Residence			
Rural	Reference	1	
Urban	1.30	1.05-3.59	<0.001
Job			
Unemployment	Reference	1	
Employed	0.66	0.47-0.87	0.004
Educational level			
Illiterate	Reference	1	
Primary school	0.89	0.73-1.13	0.093
Guidance/high school	0.82	0.63-0.98	0.048
Diploma	0.73	0.52-0.91	0.001
University	0.72	0.68-0.97	0.001
Marital statues			
Marriage	Reference	1	
Single	0.91	0.63-0.84	<0.001
Economic status			
Low	Reference	1	
Middle	0.86	0.67-1.37	0.291
High	0.83	0.58-0.96	0.006

OR; odds ratio, CI; confidence intervals.

**Figure 3:**
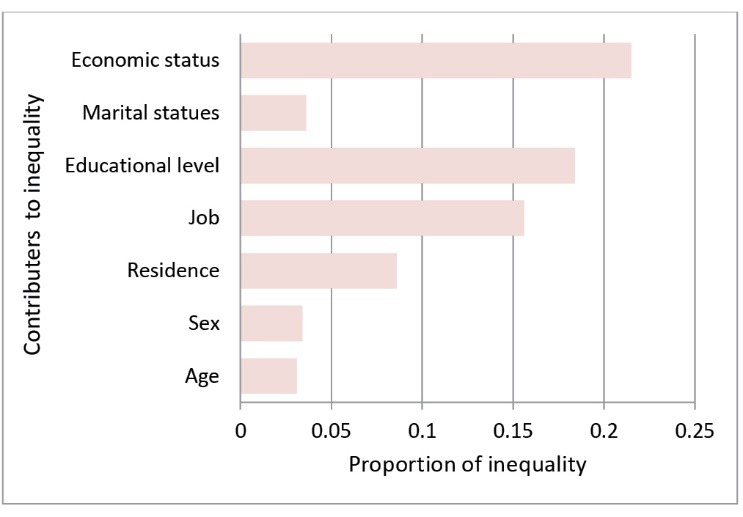
Proportion of inequality by socioeconomic contributions in high blood pressure.

Concentration index was decomposed to investigate the main socioeconomic contributors to inequality in hypertension. Age, sex, reign, educational level, job, marital status and economic status were the contributors that had significant effect on hypertension based on logistic regression model. It was found that the important socioeconomic contributors in inequality were job (*P *= 0.008), educational level (*P *= 0.005), and SES (*P *= 0.003) (Table 2). According to concentration index decomposition, the main sources of inequality in hypertension were job (15%), educational level (18%), and economic status (21%), respectively. In general, 74.2% of inequality was explained by 7 socio-economic determinants and 25.8% of inequality was due to other contributors that were not included in the study (Figure 3).

**Table 2:** The main socioeconomic contributors to high blood pressure according to decomposing of inequality line

	**t- statistic**	**P value**	**Elasticity**	**Concentration index**	**Contributions**
Age	2.64	0.088	0.30	-0.01	0.031
Sex	-2.80	0.095	-0.39	-0.02	0.034
Residence	-1.88	0.128	-0.08	0.08	0.086
Job	-2.96	0.008	-0.08	0.09	0.156
Educational level	-4.37	0.005	-0.11	0.15	0.184
Marital statues	-1.53	0.128	-0.08	0.08	0.036
Economic status	-3.36	0.003	-0.12	0.11	0.215
Residual	16.04	0.000	0.000	-	0.258
Total				-	1.0

## Discussion

A total of 690 adults were studied in Ilam province to find the magnitude of inequality in high blood pressure and to identify its contributory factors. It was found that hypertension was more prevalent in lower SES groups. The decomposition analyses also revealed that important contributory factors of inequality were job, education, and economic status. Namely, unemployment and lower educational level as well as the lower economic status had a greater contribution to the development of the inequality. This should be considered in developing efficient interventions to prevent and control high blood pressure.

Similar to our results, a study in Japan has shown that the economic status and education were associated with the dietary salt intakes, which is attributed to the development of high blood pressure. It also showed a negative association between SES and the prevalence of hypertension with a marked emphasis on the importance of education.^9^ The results of the study on Canadian population showed that hypertensive patients who were young, male, and those with lower SES were less likely to adhere to the recommended lifestyle behaviors for blood pressure control. This finding highlights the important subgroups to target for improved lifestyle management of hypertension.^2^ In most developing countries such as Iran, there is no precise index for this classification; thus, the results of such studies may be different.

Age, sex and marital status were the other positive contributors in inequality of high blood pressure. Regarding the age, it was shown that low level of SES is mostly found in the older-age groups. In this study, the sex ratio in the SES groups indicates that the lower participants in the high-economic group are women, whereas women comprise higher low-SES group. On the other hand, although there was a significant relationship between single and married individuals, this difference may be primarily due to different age structures of these groups.

The income level was not analyzed in our study due to the difficulties in obtaining the income levels by self-report methods. Therefore, education and other explanatory socio-economic factors were investigated. Thus, these factors have a strong contribution to the SES effects on health outcomes than self-reporting nature of income level.

In this study, it was found that the low SES individuals have a higher prevalence of high blood pressure that is partially explained by the patterns of food choices/purchases. Studies have shown that economically disadvantaged populations have less dietary intakes in favor of prevention of the chronic disease such as high blood pressure. Poor people are less likely to purchase healthy foods, besides the fact that they are living disproportionally in areas where their access to healthy and fresh foods is limited.^10^ Previous studies have reported that socioeconomically disadvantaged individuals are less likely to purchase foods with low salt or have engaged in salt restriction, and the intakes of total sodium and salt would decrease as educational level increases.^2,11^ Also, our study revealed that high blood pressure is more prevalent in lowest quintiles of socio-economic distribution.

Health promotion efforts to encourage people to purchase and consume ‘healthy’ and nutritious foods have been also reported to be more successful with socioeconomically advantaged groups.^11,12^ On the other hand, some researchers have argued that the impact of well‐intentioned health promotion efforts could be differential and further widen the pre-existing inequality inadvertently.^13^

Regarding the present study, blood pressure measures were extracted from records of healthcare center. It has been shown that using standardized measures of diseases would lead to an apparent concentration of the disease among lower SES groups,^14^ these findings can add to the ongoing debate on whether chronic diseases in low-to-middle income countries are really concentrated among the poorer groups compared to high-income countries.^15^

## Limitations

Some limitations should be addressed in our study, in Iran like other developing countries; there was no national index for dividing the population into different socioeconomic groups. Other limitation is that in cross-sectional studies like this study, the associations between variables should be interpreted with caution, and the associations between variables were not necessarily causal.

## Conclusion

It seems that more studies are needed to focus on food purchasing patterns and dietary behaviors of chronic patients in Iran. Moreover, diet-related health promotion and educational strategies should be integrated to the growing interest in health inequalities within populations. One substantial key point to achieve an effectiveness approach to deal with chronic diseases might be building partnership with disadvantaged populations. By doing so, specific methods will be tailored, which will be more sensitive to economical and societal barriers to adopt healthy lifestyle.

## Ethical approval

This study approved by ethical committee in the Ilam University of Medical Sciences (ID: ir.medilam.rec.1395.94).

## Competing interests

All authors declare no competing financial interests exist.

## Funding

Vice Chancellor for Research and Technology of Ilam University of Medical Sciences funded and supported the present study (ID: 957020.123).

## Acknowledgement

We would like to thank health staffs of Ilam University of Medical Sciences, which help us in data collection
